# Supplementary material for: Assessing food consumed away from home in low-and middle-income countries by developing specific modules for household surveys: Experimental evidence from Vietnam and Burkina Faso
Source: PLoS One. 2024 Dec 2;19(12):e0314786. doi: 10.1371/journal.pone.0314786 (PMC11611215; doi:10.1371/journal.pone.0314786)
Supplement: S1 File — S1 Table 1. Long list and short list, Vietnam. S1 Table 2. Long list and short list, Burkina Faso. S1 Fig 1. Bland and Altman plots for Vietnam: A) for protein; B) for carbohydrate; C) for lipid. 24HDR: 24-hour dietary recall. S1 Fig 2. Bland and Altman plots for Burkina Faso: A) for protein; B) for carbohydrate; C) for lipid. 24HDR: 24-hour dietary recall. (DOCX) [file pone.0314786.s002.docx]

**Supplementary materials**

**S1 Table 1. Long list and short list, Vietnam**

| **Long list** | **Short list** |
| --- | --- |
| Alcohol (beer, wine, …) | Alcohol (beer, wine,…) |
| Fruit juice, fresh | Non alcoholic beverages |
| Hot/ice beverage (tea, coffee, …) with sugar and milk |  |
| Hot/ice beverage (tea, coffee, …), plain |  |
| Fruit smoothies |  |
| Sugar sweetened beverages (fruit juice, soda, …) |  |
| Eggs | Eggs |
| Tofu (boiled) | Tofu dish |
| Tofu (fried) |  |
| Fish cooked/boiled/baked/steamed any kind of cooking method but frying | Any kind of fish, seafood, fresh water food whatever the cooking |
| Fish/fried |  |
| Fish, dried |  |
| Seafood, cooked/boiled/baked/steamed any kind of cooking method but frying |  |
| Seafood, fried |  |
| Seafood, dried |  |
| Shrimp, water fresh, fried/stir fried |  |
| Poultry, cooked/braised/boiled/roasted/steamed/baked any kind of cooking method but frying | Any kind of meat including offals |
| Poultry, fried |  |
| Meat (beef, veal, pork), cooked/braised/boiled/roasted/steamed/baked any kind of cooking method but frying |  |
| Meat (beef, veal, pork), dried |  |
| Meat (beef, veal, pork), fried |  |
| Offals, cooked/ fried |  |
| Offals, cooked/boiled |  |
| Pork ear with rice powder |  |
| Meat proccessed | Meat proccessed |
| Seafood with eggs, cooked/braised | Any kind of animal products, mixed |
| Pork with eggs, cooked/braised |  |
| Pork with shrimp, stir fried |  |
| Tofu dish with meat |  |
| Meat dish with vegetables |  |
| Vegetable dish with eggs |  |
| Offals dish with vegetables |  |
| Seafood and vegetable, stir fried |  |
| Mix salad, with poultry or meat |  |
| Mix salad, with shrim of fish/seafood |  |
| Mix salad, with both (meat/shrim of fish/seafood/tofu…) |  |
| Meat with fried potatoes | Meat with fried potatoes |
| Corn/sweet potato/potato boiled | Corn/potato/sweet potato, boiled |
| Corn/potato/sweet potato, fried | Corn/potato/sweet potato, fried |
| Fried rice, mixed | Rice based side dish |
| Rice, plain, any kind |  |
| Rice cake |  |
| Sticky rice cake (sweet) |  |
| Sticky rice cake (salty) |  |
| Sticky rice with meat |  |
| Sticky rice with nuts and/or beans |  |
| Green salad | Vegetables or salad |
| Vegetables cooked/steamed/boiled |  |
| Vegetables, fried/stir fried |  |
| Office meal no meat no fish, with vegetables | Office meal with no animal products |
| Office meal with fish or/and sea food | Office meal with any kind of animal products |
| Office meal with meat |  |
| Office meal (mix meat &fish/tofu/egg…) |  |
| Meat hotpot | Hot pot |
| Poultry hotpot |  |
| Seafood hotpot |  |
| Mixed hotpot |  |
| All kind of roll (mix meat & vegetables)/NOT fried | Roll |
| All kind of roll (mix meat & vegetables)/ fried |  |
| Rice soup, without any animal product | Rice soup with any kind of animal products |
| Rice soup with fish |  |
| Rice soup with meat/ offals |  |
| Rice soup with poultry |  |
| Rice soup with seafood |  |
| Rice soup with nuts |  |
| Rice soup, mixed (meat, seafood, nuts…) |  |
| Soup with meat | Soup with any kind of animal products |
| Soup with poultry |  |
| Soup with seafood |  |
| Vegetables soup with fish | Vegetables soup with any kind of animal products |
| Vegetables soup with meat or poultry |  |
| Vegetables soup with tofu |  |
| Vegetables soup with eggs |  |
| Vegetables soup mixed (both meat &fish/tofu/egg…) |  |
| Vegetables soup without meat/fish… | Vegetables soup with no animal product |
| Noodle, vermicelli, pasta with soup (with meat/offals or poultry) | Noodle, vermicelli, pasta, with soup, with any kind of animal product |
| Noodle, vermicelli, pasta with soup (with fish or seafood) |  |
| Noodle, vermicelli, pasta with soup (with tofu) |  |
| Noodle, vermicelli, pasta with soup (with eggs) |  |
| Noodle, vermicelli, pasta with soup, mixed (with meat/offals & poultry& seafood& tofu...) |  |
| Instant noodle with soup, with any kind of animal product |  |
| Noodle, vermicelli, pasta without soup (with meat/offals or poultry) | Noodle, vermicelli, pasta, without soup, with any kind of animal product |
| Noodle, vermicelli, pasta without soup (with fish or seafood) |  |
| Noodle, vermicelli, pasta without soup (with tofu) |  |
| Noodle, vermicelli, pasta without soup (with eggs) |  |
| Noodle, vermicelli, pasta without soup, mixed (with meat/offals & poultry& seafood& tofu...) |  |
| Instant noodle without soup, with any kind of animal product |  |
| Sandwich | Sandwich, Hamburgers |
| Hamburger |  |
| Pizza | Pizza |
| Salty snack (peanut, potato crips…) | Salty snacks |
| Sweets and candies | Sweets, sugary snacks |
| Ice-cream |  |
| Sweet gruel |  |
| Cereal cake/powders |  |
| Nuts | Nuts |
| Milk and milk substitutes | Dairies |
| Milk condensed sweetened |  |
| Yogurt |  |
| Fruit, any kind | Fruit |

**S1 Table 2. Long list and short list, Burkina Faso**

| **Long list** | **Short list** |
| --- | --- |
| Cashew nuts | Nuts and peanuts |
| Peanut |  |
| Wheat flour cake | Cake and donut |
| Bean fritter |  |
| Chips | Chips |
| Biscuit, sweet | Biscuit, pastries and pancakes |
| Packaged cookies |  |
| Galette (wheat, millet, bean), fried, sweet |  |
| Galette (millet), fried, sweetened with yeast |  |
| Fried bean pancake, sweetened with yeast |  |
| Croissant, pastries |  |
| Cereal porridge (rice, corn, millet, etc.) | Cereal porridge (rice, corn, millet, etc.) |
| Pineapple, orange, apple and guava | Fruits |
| Papaya, mango |  |
| Banana |  |
| Chocolates | Chocolates, sweets |
| Candy |  |
| Yogurt |  |
| Ice cream |  |
| Black tea, green tea, lipton, coffee, sugar free | Black tea, green tea, lipton, coffee, with sugar and/or milk |
| Black tea, green tea, lipton, coffee, sweet |  |
| Black tea, green tea, lipton, coffee, without sugar, with milk |  |
| Black tea, green tea, lipton, coffee, sweetened, with milk |  |
| Candy, cola, fanta and sugar water | Sweet drinks |
| Fresh and industrial fruit juice, bissap juice, tamarind juice and ginger juice | Fruit juice, ginger juice, tamarind or bissap |
| Zoom-koom (made from millet flour) |  |
| Industrial beer | Alcoholic drinks |
| Dolo |  |
| Wine, sangria |  |
| Liqueur (JB, Chivas, etc.) |  |
| Skewer (beef, mutton, goat, etc.) | Braised or grilled meat |
| Grilled/baked meat (beef, mutton, goat, etc.) |  |
| Braised or grilled chicken |  |
| Braised liver, grilled offal |  |
| Merguez, sausages |  |
| Fried chicken | Fried chicken |
| Fish skewer | Grilled fish or shellfish |
| Grilled fish |  |
| Fried fish | Fried fish |
| Pizza | Pizza |
| Hamburger | Hamburger |
| Omelette | Omelette |
| Omelette with bread (sandwich with egg) | Sandwich |
| Avocado sandwich |  |
| Meat sandwich (minced meat, liver, etc.) |  |
| Vegetable with egg sandwich |  |
| Condensed milk and bread |  |
| Fried potatoes | Yam, potato, sweet potato and fried plantain |
| Sweet potatoes, fries |  |
| Yam, fries |  |
| Alloco (plantain), fried |  |
| Boiled or steamed potatoes | Boiled yam, potato and sweet potato |
| Sweet potatoes, boiled |  |
| Yam, boiled |  |
| Bread alone |  |
| Raw vegetables salad with vinaigrette | Raw vegetable salad with vinaigrette |
| Meat soup | Meat or fish soup |
| Fish soup |  |
| Fat rice | Fatted rice, pasta with fat, yam ragout, without fish or meat |
| Yam stew |  |
| Pasta (macaroni, spaghetti) with fat |  |
| Fat rice with meat, soumbala rice | Fatted rice with meat, soumbala rice |
| Fatty rice with fish | Fatted rice with fish |
| Prepared beans, with millet and oil | Beans prepared with rice, maize, millet or couscous |
| Prepared beans, with rice and oil |  |
| Prepared beans, with corn and oil |  |
| Prepared beans, with oil | Beans on their own, Gonré, Potatoes |
| Gonré prepared plain |  |
| Boiled ground peas/suma |  |
| Babenda (leaf stew and peanut rice) |  |
| White/yellow maize toast with fresh okra sauce or sorrel sauce or leafy vegetable sauce, without fish or meat | Corn toast with sauce, without fish or meat |
| White/yellow maize toast with fresh okra sauce or sorrel sauce or leafy vegetable sauce, with meat | Corn toast with sauce and meat |
| White/yellow maize toast with fresh okra sauce or sorrel sauce or leafy vegetable sauce, with fish | Maize toast with sauce and fish |
| White rice with peanut paste sauce, without fish or meat | Rice, pasta, fonio, foutou or couscous with sauce, without fish or meat |
| White rice with seed sauce without fish or meat |  |
| White rice, pasta or couscous with tomato sauce without fish or meat |  |
| White rice with leafy vegetable sauce or okra sauce without fish or meat |  |
| White rice with yassa sauce without fish or meat |  |
| White rice with clear sauce without fish or meat |  |
| Attiéké oil onion raw vegetables |  |
| Foutou yam or banana with seed sauce without fish or meat |  |
| Foutou yam or banana with clear sauce without fish or meat |  |
| Fonio with leaf sauce without fish or meat |  |
| Fonio with peanut paste sauce without fish or meat |  |
| White rice with peanut paste sauce and meat | Rice, pasta, fonio, foutou or couscous with sauce and meat |
| White rice with seed sauce and meat |  |
| White rice, pasta or couscous with tomato sauce and meat |  |
| White rice with leafy vegetable or okra sauce and meat |  |
| White rice with yassa sauce and meat |  |
| White rice with clear sauce and meat |  |
| Foutou yam or banana with seed sauce and meat |  |
| Foutou yam or banana with clear sauce and meat |  |
| Fonio with leaf sauce and meat |  |
| Fonio with peanut paste sauce and meat |  |
| White rice with peanut paste sauce and fish | Rice, pasta, fonio, foutou or couscous with sauce and fish |
| White rice with seed sauce and fish |  |
| White rice, pasta or couscous with tomato sauce and fish |  |
| White rice with leafy vegetable or okra sauce and fish |  |
| White rice with yassa sauce and fish |  |
| White rice with clear sauce and fish |  |
| Foutou yam or banana with seed sauce and fish |  |
| Foutou yam or banana with clear sauce and fish |  |
| Fonio with leaf sauce and fish |  |
| Fonio with peanut paste sauce and fish |  |

| A | Protein | 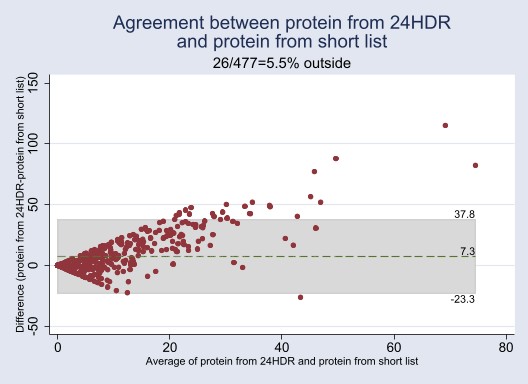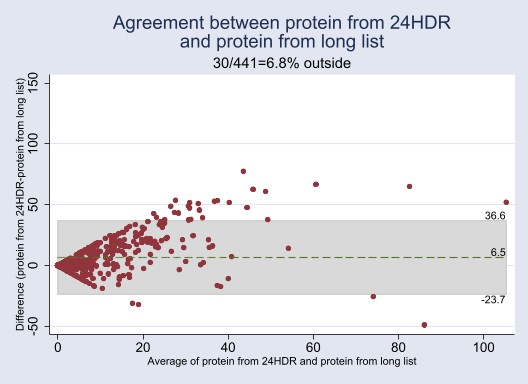 |
| --- | --- | --- |
| B | Carbohydrate | 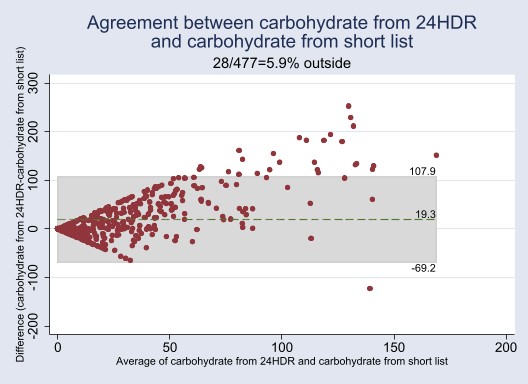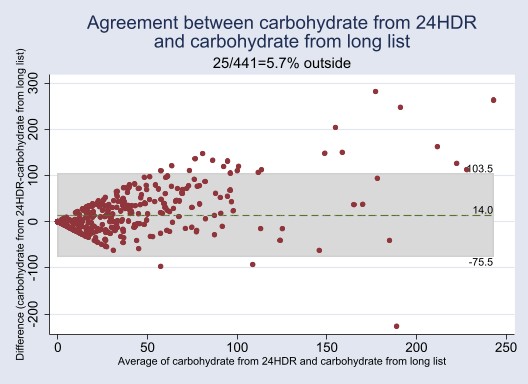 |
| C | Lipid | 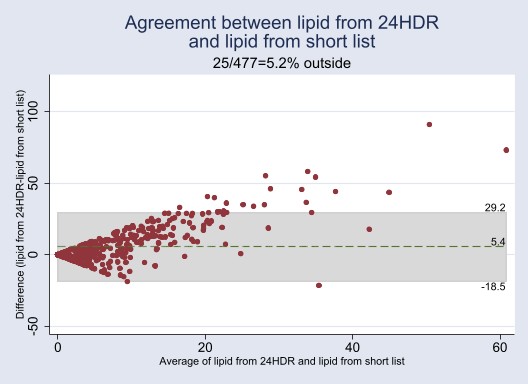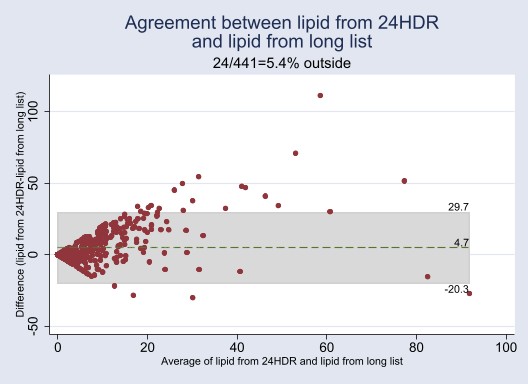 |

**S1 Figure 1. Bland and Altman plots for Vietnam: A) for protein; B) for carbohydrate; C) for lipid.** 24HDR: 24-hour dietary recall.

| A | Protein | 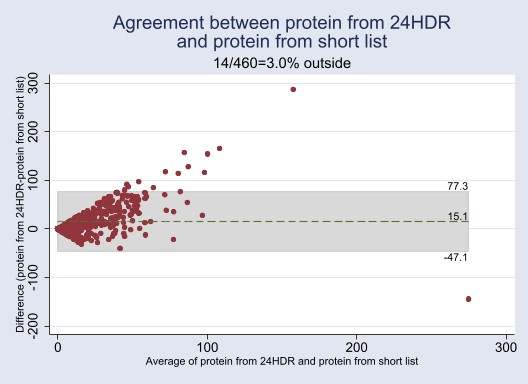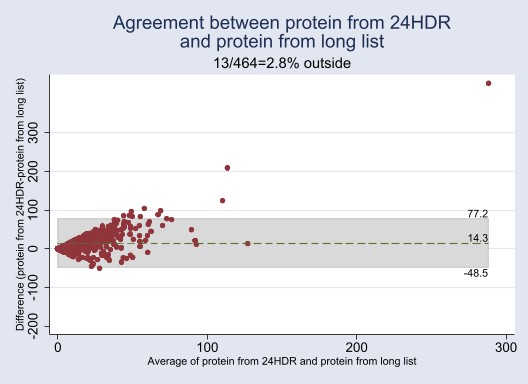 |
| --- | --- | --- |
| B | Carbohydrate | 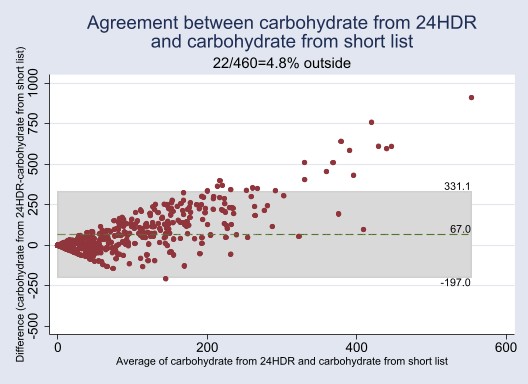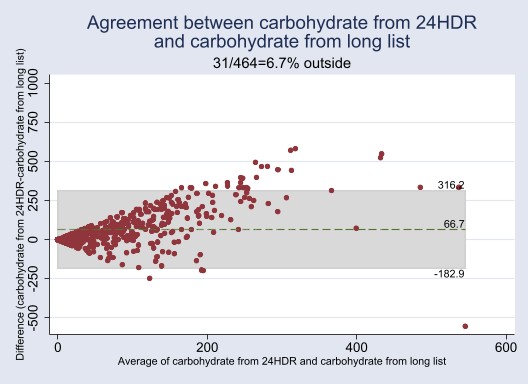 |
| C | Lipid | 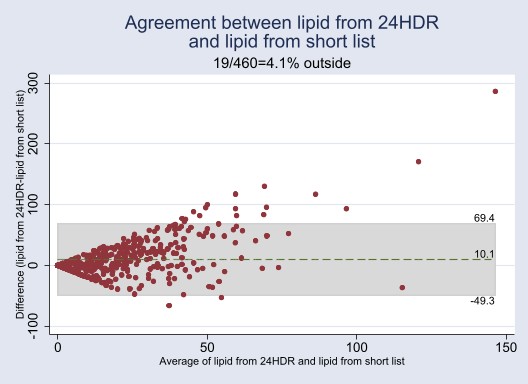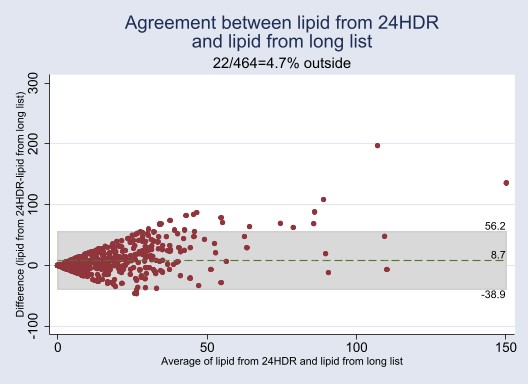 |

**S1 Figure 2. Bland and Altman plots for Burkina Faso: A) for protein; B) for carbohydrate; C) for lipid.** 24HDR: 24-hour dietary recall.
